# Laparoscopy Combined with Enhanced Recovery Pathway in Ileocecal Resection for Crohn's Disease: A Randomized Study

**DOI:** 10.1155/2018/9648674

**Published:** 2018-11-11

**Authors:** Yibin Zhu, Jianjian Xiang, Wei Liu, Qian Cao, Wei Zhou

**Affiliations:** ^1^Department of General Surgery, Sir Run Run Shaw Hospital, School of Medicine, Zhejiang University, Hangzhou, China; ^2^Department of Gastrointestinal Surgery, Longyan First Hospital, Fujian Medical University, Longyan, China; ^3^Department of General Surgery, Ningbo Second Hospital, Ningbo, China; ^4^Inflammatory Bowel Disease Center, Sir Run Run Shaw Hospital, School of Medicine, Zhejiang University, Hangzhou, China; ^5^Department of Gastroenterology, Sir Run Run Shaw Hospital, School of Medicine, Zhejiang University, Hangzhou, China

## Abstract

**Background and Aims:**

Laparoscopic approach is recommended as the first-choice option for simple ileocecal resections. However, there are no randomized trials that have focused on patients with Crohn's disease (CD) treated by laparoscopy and enhanced recovery pathway. The aim of the present study is to prospectively evaluate the feasibility, safety, and short-term outcomes of laparoscopy with enhanced recovery pathway for CD patients undergoing ileocecal resection.

**Methods:**

A consecutive cohort of 32 CD patients who underwent laparoscopic ileocecal resection between December 2015 and December 2016 was randomized to enhanced recovery after surgery (ERAS) group or standard care group. Primary outcome was total postoperative hospital stay. Secondary outcomes were time to first flatus and stool, pain scores, morbidity, reoperation rate, readmission rate, and in-hospital costs.

**Results:**

Compliance with the ERAS was high for all items (≥90%) except the items of abdominal drains and early fluid intake. A significantly earlier return of bowel function was observed in the ERAS group. Compared with the standard care group, patients in the ERAS group had shorter postoperative hospital stay and lower in-hospital costs (5.19 ± 1.28 versus 9.94 ± 3.33 days, *P* < 0.001; 2.70 ± 0.50 versus 3.73 ± 0.75 ten thousand RMB, *P* < 0.001, respectively). Other parameters did not show any significant differences between the two groups.

**Conclusions:**

Laparoscopic approach within an ERAS perioperative care program is a safe and effective treatment combination for CD patients requiring ileocecal resection. This study is registered at ClinicalTrials.gov (NCT02777034).

## 1. Introduction

Crohn's disease (CD) is a chronic inflammatory bowel disease affecting any part of the gastrointestinal tract, with the terminal ileum being the most frequently involved site [1]. In spite of the significant advances in medical therapy, the lifetime likelihood of surgery is estimated to be 70~90% [2, 3]. The laparoscopic approach has been validated for surgical resection of nonfistulizing CD with a faster recovery of bowel movements and normal diet as well as reduced postoperative morbidity and a shorter hospital stay in comparison with open surgery [4]. The laparoscopic approach is currently recommended as the first-choice option for simple ileocecal resections [5]. However, the overall benefits of laparoscopic surgery may be counteracted by traditional care regimens, which even cannot have an advantage over the open procedures combined with accelerated recovery care programs [6, 7].

Enhanced recovery after surgery (ERAS) program was first developed by Henrik Kehlet in the 1990s, with the aim of reducing surgical stress and achieving faster recovery for patients [8]. ERAS has shown advantages in the postoperative outcomes of patients undergoing open or laparoscopic resection for gastrointestinal cancer [9, 10]. The use of laparoscopic surgery combined with ERAS programs in CD is calling for a reassessment. Theoretically, the combination of minimally invasive surgery with a subsequent reduced surgical stress responses should provide a rational basis for an even faster recovery. Unfortunately, to date, there is limited evidence on the use of ERAS in patients with CD [11, 12], even few evidence on the use of laparoscopy combined with ERAS. Only a single case-matched study showed a significantly faster return to normal bowel function and shorter hospital stay for patients with primary ileocecal CD undergoing laparoscopic surgery and ERAS pathway [12]. There are no randomized reports that have focused on this topic. The aim of the present study is, therefore, to prospectively evaluate the feasibility, safety, and short-term outcomes of laparoscopy with ERAS for CD patients undergoing ileocecal resection.

## 2. Methods

### 2.1. Patient Population and Study Design

From December 2015 to December 2016, this study was prospectively performed at SRRSH Hospital, Zhejiang University Hospital. Patients were eligible if they were between 14 and 70 years of age, had histologically proven CD with disease localized to the terminal ileum with or without cecum involvement. Exclusion criteria were previous bowel resection, evidence of abscesses or fistulas, emergency surgery, contraindications to laparoscopy, or a planned stoma. The study was conducted in accordance with the principles of the Declaration of Helsinki. The independent medical ethics review boards of SRRSH hospital approved the study protocol. This study is registered at ClinicalTrials.gov (NCT02777034).

Patients were randomized by means of an internet randomization module to ERAS care group or standard care group. All patients were informed about the procedure and the perioperative management and provided written informed consent but were blinded to the type of group, i.e., ERAS care or conventional care. Perioperative protocols in the two treatment groups are summarized in Table 1.

Postoperative analgesia consisted of administration of Parecoxib Na 40 mg intravenously (i.v.) every 6–8 h. When oral intake was possible, analgesia regimen was ibuprofen 600 mg (no more than 1.2 g/day) if visual analog scale (VAS) > 4. The postoperative regimen has been described elsewhere [13]. In the ERAS group, gastrointestinal tubes were not used and postoperative mobilization and oral intake started from the day of operation. The urinary bladder catheter was removed routinely 24 h postoperatively. All patients were discharged if they complied with the following predefined discharge criteria: (1) adequate pain control with oral analgesics; (2) ability to tolerate solid food; (3) passage of first flatus and/or first stool; and (4) mobilization as preoperative.

### 2.2. Surgical Technique

Laparoscopic ileocecal resection was performed with a standardized technique; three trocars were placed (one 10 mm trocar in the umbilical area, one 12 mm trocar in the left upper flank, and one 5 mm trocar in the left lower flank), while an auxiliary 5 mm trocar was placed in the right flank if further retraction was needed. A modified lithotomy position in steep Trendelenburg position with the left side down was then adopted. The small and large bowels were examined to exclude other lesions; the distal ileum and right colon, including the hepatic flexure, were dissected using a lateral to median approach, and the ileocecal region was exteriorized through a short midline extension of the periumbilical access. Extension to more than 7 cm was defined as conversion to open. Mesentery division, resection of the affected bowel with a 2 cm macroscopically normal margin, and a stapled functional end-to-end anastomosis were performed extracorporeally. The surgical technique used was the same in both treatment groups.

### 2.3. Data Collection and Outcome

Preoperative, operative, and postoperative data were prospectively recorded for each patient of both groups. Preoperative data included age, sex, body mass index (BMI), disease pattern, American Society of Anesthesiologists (ASA) score, and preoperative medical therapy. Operative data included duration of surgery, intraoperative complications, and additional intraoperative details. Primary outcome was total postoperative hospital stay. Total postoperative hospital stay was defined as postoperative hospital stay plus the additional hospitalization period in case patients were readmitted within 30 days of surgery. Secondary outcomes were time to first flatus and stool, pain scores (according to the VSA), overall morbidity (according to the Dindo–Clavien classification) [14], reoperation rate, readmission rate, infectious complication rate within 30 days of hospital discharge, in-hospital mortality, and in-hospital costs. The in-hospital costs included the costs of clinical examination, surgery, nursing, and medications as well as the costs of complications, reoperations, and readmissions within 30 days after the index operation. Compliance with the major items of the ERAS was assessed.

### 2.4. Statistical Analysis

Data were analyzed in accordance with the intention to treat principle. Data were presented as means ± standard deviations or as medians and interquartile ranges where appropriate. Chi-squared tests were used to compare categorical data. The Mann–Whitney *U* test was used for continuous not normally distributed outcomes. For continuous normally distributed data, the independent sample *t*-test was used. A 2-sided *P* value < 0.05 was considered to be statistically significant. All statistical analyses were performed using SPSS version 18.0 (SPSS Inc., Chicago, IL).

## 3. Results

**Figure 1:**
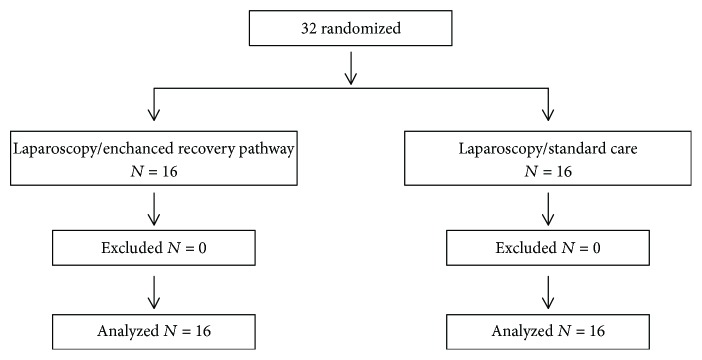
Study flow chart.

Between December 2015 and December 2016, 32 consecutive patients were enrolled in the study. 16 patients were treated with conventional care, 16 with ERAS pathway (Figure 1). Patient characteristics are summarized in Table 2. All patients underwent laparoscopic ileocecal resection with standardized technique with no conversion to open surgery. A covering stoma was not performed in any patient.

### 3.1. ERAS Protocol Compliance

Compliance with the ERAS was quite high, reaching 90% or more for all items, with the exception of avoidance of abdominal drains and early fluid intake on postoperative day 0. One ERAS patient failed to early mobilization on postoperative day 1 because of dizziness. One ERAS patient placed abdominal drains because of hypoalbuminemia and higher C-reactive protein level; another patient placed abdominal drains due to damage of the hepatic flexure during the operation. Two ERAS patients took in fluid on postoperative day 1 because of nausea. The compliance rate with the major items of enhanced recovery pathway is presented in Table 3.

### 3.2. Short-Term Outcomes

Postoperative outcomes of the two groups are reported in Table 4. A significantly earlier return of bowel function (time to first flatus and time to stool passage) was observed in the ERAS group compared with conventional care. Compared with the standard care group, patients in the ERAS group had shorter postoperative hospital stay and lower in-hospital costs (5.19 ± 1.28 versus 9.94 ± 3.33 days, *P* < 0.001; 2.70 ± 0.50 versus 3.73 ± 0.75 ten thousand RMB, *P* < 0.001, respectively). Other parameters did not show any statistically significant differences between the two groups.

No mortality occurred during the study period. No major complications occurred in either the ERAS group or the conventional care group. Minor complications included two prolonged postoperative ileus (grade I) in the ERAS group and two wound infections (all grade I) in the conventional care group. One rehospitalization (in community hospital) within 30 days after discharge occurred in the ERAS group because of unspecific abdominal pain. No infectious complications occurred in either the ERAS group or the conventional care group within 30 days after discharge.

## 4. Discussion

The European Crohn's and Colitis Organization (ECCO) and European Society of Coloproctology (ESCP) have recommended to apply the principles of the ERAS program for CD [5]. However, to date, there are no randomized reports that have focused on the combination of ERAS with laparoscopic surgery in patients with CD. Our results support the safety of an ERAS protocol in laparoscopic ileocecal resection for CD and demonstrate its efficacy in reducing length of stay, time to bowel function, and in-hospital costs.

The terminal ileum and cecum are frequently involved in CD, making ileocecectomy among the most common surgical interventions performed in this population who are not responding to conventional treatment. The first description of a laparoscopic intestinal resection for CD was reported in 1993 by Milsom et al. [15]. Since then, many studies have evaluated the use of the laparoscopic surgery in CD. It had a significantly quicker recovery of bowel function and reduced postoperative morbidity rates, length of hospital stay, and rates of postoperative intestinal obstruction [16, 17]. Some researchers have also demonstrated excellent long-term results of laparoscopic ileocecal resection for CD [18–20]. Based on these findings, laparoscopy is currently recommended as the surgical method of choice for simple ileocecal resection according to the ECCO-ESCP consensus [5].

Theoretically, the combination of ERAS care and laparoscopy surgery will result in the fastest postoperative recovery. At the same time, it is questionable if both of them are as important in postoperative recovery. In our study, we chose to standardize our patient population to those undergoing a single laparoscopic procedure in order to decrease confounding variables and to separate the effect of ERAS management from that of the minimally invasive approach alone. Overall, ERAS patients realized benefits in terms of length of stay, time to bowel function, and narcotic use, without any statistically significant increase in complications. The decrease in hospital stay provides benefit in terms of cost savings and potentially fewer lost work hours for family members, while offering improved patient comfort, reducing exposure to hospital-acquired infections, and decreasing social isolation among these chronically ill adolescents.

In our study, the in-hospital costs were significantly lower in the ERAS group, which were most likely caused by a shorter hospital stay, because the overall morbidity are similar between the two groups. However, in LAFA study, in-hospital costs were similar between the two groups. That is because laparoscopy as well as fast-track care is more expensive than open surgery and standard care [9]. In our hospital, usually no additional costs of ERAS care happened.

Patient and family education is critical to the success of any ERAS program and perhaps even more so in the setting of CD when patients may have endured prolonged or multiple hospitalizations. During preoperative discussion, patients should be counseled regarding the postoperative management plan and expected length of stay to ensure that their early discharge is not perceived as rushed or premature. Furthermore, appropriate education must be provided regarding potential complications and concerning symptoms in order to avoid any delay in presentation.

The results of this prospective randomized study in patients undergoing ileocecal resections for CD with ERAS care confirm the results with similar fast-track regimens in elective colonic resections in patients with noninflammatory bowel disease [9]. Our results are different from those published before with laparoscopic-assisted ileocolic resections for CD [16, 17, 21], since most of the studies have focused on confirming the benefits of laparoscopic approach over open surgery in patients with CD. However, the overall benefits of laparoscopic surgery may be counteracted by traditional care regimens. Similar or even faster rates of recovery have been reported for open colectomy combined with accelerated recovery care programs in comparison with laparoscopic colectomy in a standard care setting [6, 9]. Thus, pointing out the effect of ERAS care in laparoscopic ileocecal resection for CD is necessary. However, there is limited evidence on the use of ERAS care in CD patients with laparoscopic ileocecal resection. Only a single case-matched study has reported the feasibility and safety of ERAS pathway for patients with primary ileocecal CD undergoing laparoscopic surgery [12]. To the best of our knowledge, our study represents the first randomized trial reported experience of laparoscopy with ERAS in CD patients, although the number of patients is small, and supports the results that laparoscopic approach within an ERAS perioperative care program is the optimal treatment combination for CD patients requiring ileocecal resection.

Parecoxib was routinely used in order to decrease narcotic use in fast-tracked patients. Many alternative pain management strategies have been described in fast-track surgery, including loco-regional anesthesia and epidural or spinal anesthesia as well as nonnarcotic adjuncts including nonsteroidal anti-inflammatory drugs. This class of medications raises particular issues in the context of CD, as NSAIDs have been associated with onset or relapse of colitis in patients with newly diagnosed or chronic inflammatory bowel disease [22]. Some recent literature in adult patients has begun to refute this notion [23]. In our limited sample, we saw no increase in subsequent disease activity in patients who received perioperative ketorolac, but the current study lacks sufficient power to exclude any possible association.

The limitations of our study were the nonblinding of the treatment. Second, the number of patients is relatively small. Obviously, further larger prospective study is required to assess the safety and efficacy of such an approach in laparoscopic ileocecal resection for CD. Third, the effect of ERAS combined with laparoscopy on long-term outcome like disease recurrence should be observed in the long run. Further analysis on improvements in clinical outcome related to improved experience and compliance with the ERAS protocol will be obtained in the future.

In conclusion, this study shows that optimized perioperative care combined with minimally invasive techniques leads to further improvements in surgical outcomes for CD patients. The optimal treatment combination for patients requiring ileocecal resection for CD is a laparoscopic approach within an ERAS perioperative care program.

## Figures and Tables

**Table 1:** Perioperative programs in the two treatment groups.

Enhanced recovery after surgery program	Conventional care
*Preoperative*	*Preoperative*
Multidisciplinary patient information	Patient information
No bowel preparation	Mechanical bowel preparation
No fasting, fluids until 2 h before surgery, solids until 6 h	Fasting since midnight before operation
Orally take 1000 mL + 500 mL 5% glucose solution the night before and on the morning of surgery	No 5% glucose solution
*Intraoperative*	*Intraoperative*
Laparoscopic standardized technique	Laparoscopic standardized technique
Fluid restriction (max 1500 mL)	Fluid overload (over 1500 mL)
Prevention of deep vein thrombosis: stretch socks	No stretch socks
Infusion heating	No infusion heating
No abdominal drainage	Abdominal drainage
*Postoperative*	*Postoperative*
No nasogastric tube removal at awakening	Nasogastric tube removal after passing flatus
Early mobilization 2 h after surgery	Mobilization from postoperative day 1
Early diet intake, fluids in postoperative day 0, and soft food in postoperative day 1	Fluids and solids intake after first passage of stools
Opioid-free analgesia	Opioid-free analgesia
Urinary catheter removal on postoperative day 1	Urinary catheter removal on postoperative day 2/3

**Table 2:** Baseline characteristics and surgical aspects of the included patients per group.

	Laparoscopy and ERAS care (*n* = 16)	Laparoscopy and standard care (*n* = 16)	*P* value
Age, year, median (IQR)	31.5 (29.25, 43.50)	29.5 (26.25, 43.50)	NS
Course of disease, month median (IQR)	32 (12, 81)	42 (10.5, 81)	NS
Male sex, *n* (%)	9 (56.3%)	11 (68.8%)	NS
Body mass index, mean (SD, kg/m^2^)	18.09 ± 2.35	17.68 ± 1.81	NS
ASA (%), grade I or II	100	100	NS
Smoking history, *n* (%)	2 (12.5)	5 (31.3)	NS
Immunosuppressant therapy within 30 days of surgery, *n* (%)	0 (0)	2 (12.5)	NS
Steroid therapy within 30 days of surgery, *n* (%)	0 (0)	1 (6.3)	NS
Operation history, *n* (%)	7 (43.8)	7 (43.8)	NS
Perianal disease, *n* (%)	2 (12.5)	3 (18.8)	NS
Location, *n* (%)			NS
L1 (ileal)	13 (81.3)	12 (75)	
L2 (colonic)	0	0	
L3 (ileocolonic)	3 (18.8)	4 (25)	
Laboratory indices			
White blood cell, mean, (SD, ×10^9^/L)	5.66 ± 1.75	6.21 ± 2.76	NS
Hemoglobin, g/L, median (IQR)	11.4 (10.58, 12.98)	12.1 (11.68, 13.78)	NS
C-reactive protein, mg/L, median (IQR)	2.55 (1.025, 10.45)	3.6 (1.15, 13.325)	NS
ESR, mm/h, median (IQR)	9 (6.25, 16)	9 (6, 14.75)	NS
Albumin, g/L, median (IQR)	36.85 (34.15, 38.225)	33.65 (32.15, 32.375)	NS
Preoperative thirst, *n* (%)	1 (6.3)	14 (87.5)	<0.001
Preoperative hunger, *n* (%)	1 (6.3)	14 (87.5)	<0.001
Conversion to open, *n* (%)	0	0	NS
Duration of surgery, minutes, median (IQR)	162.5 (131.25, 180)	180 (152.5, 240)	NS
Blood loss, mL, median (IQR)	30 (20, 37.5)	30 (22.5, 55.5)	NS

ERAS = enhanced recovery after surgery; IQR = interquartile range; SD = standard deviation; ASA = American Society of Anesthesiologists; ESR = erythrocyte sedimentation rate; NS = not significant.

**Table 3:** Compliance with the major items of enhanced recovery pathway.

Enhanced recovery pathway item	Compliance (%)
No bowel preparation	100%
No preoperative fasting	100%
Perioperative fluid restriction	100%
Infusion heating	100%
No nasogastric tube removal at awakening	100%
No abdominal drainage	87.5%
Early fluid intake within 6 hours after surgery	87.5%
Early mobilization on postoperative day 1	93.75%
Urinary catheter removal on postoperative day 1	100%

**Table 4:** Postoperative data in 32 patients undergoing primary ileocecal resection for Crohn's disease.

	Laparoscopy and ERAS care (*n* = 16)	Laparoscopy and standard care (*n* = 16)	*P* value
Passage of first flatus mean (SD, day)	1.75 ± 0.58	3.13 ± 0.89	<0.001
Passage of first stool mean (SD, day)	2.25 ± 1.0	4.06 ± 1.29	<0.001
Eating liquid mean (SD, day)	1.44 ± 0.63	4.38 ± 1.41	<0.001
Eating semifluid mean (SD, day)	2.75 ± 0.58	6.31 ± 1.45	<0.001
Postoperative hospital stay, mean (SD, day)	5.19 ± 1.28	9.94 ± 3.33	<0.001
Overall morbidity < 30 days, *n* (%)	2 (12.5)	2 (12.5)	NS
Complication grade I, *n* (%)	2	2	NS
Complication grade II-IV, *n* (%)	0	0	NS
Reoperations, *n* (%)	0	0	NS
Readmission < 30 days, *n* (%)	0	0	NS
Postoperative pain, VAS > 3 on day 1, *n* (%)	1 (6.3)	4 (25)	NS
Postoperative pain, VAS > 3 on day 2, *n* (%)	0	0	NS
In-hospital mortality, *n* (%)	0	0	NS
In-hospital costs (ten thousand RMB)	2.70 ± 0.50	3.73 ± 0.75	<0.001
Infectious complication within 30 days after discharge, *n* (%)	0	0	NS

ERAS = enhanced recovery after surgery; SD = standard deviation; VSA = visual analog scale; NS = not significant.

## Data Availability

The data used to support the findings of this study are available from the corresponding author upon request.
